# Supplementary material for: Chiroptical Spectroscopy, Theoretical Calculations, and Symmetry of a Chiral Transition Metal Complex with Low-Lying Electronic States
Source: Molecules. 2025 Feb 10;30(4):804. doi: 10.3390/molecules30040804 (PMC11858395; doi:10.3390/molecules30040804)
Supplement: Supplementary file 1 [file molecules-30-00804-s001.zip › molecules-3441939-supplementary.pdf]

molecules

Supplementary Material

Chiroptical Spectroscopy, Theoretical Calculations, and Symmetry of a Chiral Transition Metal Complex with Low-Lying Electronic States

Mutasem Alshalalfeh [1] and Yunjie Xu [1,*]

1 Department of Chemistry, University of Alberta, Edmonton, AB T6G 2G2, Canada; alshalal@ualberta.ca (M.A.).

* Correspondence: yunjie.xu@ualberta.ca; Tel.: +1-780-492-1244

Contents

Figure S1. The molecular formula of the (*R*,*R*)-Mn(III)-Cl-salen-chxn complex S2

Figure S2. Experimental mass spectrum of (*R*,*R*)-Co(II)-salen-chxn S2

Figure S3. The optimized geometries of the four most stable Mn(III)-Cl-salen-chxn conformers at the B3LYP-D3BJ/6-311++G(d,p) level of theory with the PCM of $CDCl_3$ S3

Figure S4. The simulated individual and Boltzmann average UV-Vis and ECD spectra of Co(II)-salen-chxn (low-spin with spin multiplicity of 2) using the B3LYP-D3BJ/6-311++G(d,p)/PCM(acetonitrile) level of theory S4

Figure S5. The simulated individual and Boltzmann average UV-Vis and ECD spectra of Co(II)-salen-chxn (high-spin with spin multiplicity of 4) using the B3LYP-D3BJ/6-311++G(d,p)/PCM(acetonitrile) level of theory S5

Figure S6. Comparison of the experimental, simulated individual and Boltzmann averaged IR and VCD spectra of Co(III)-salen-chxn at the B3LYPD3BJ/6-311++G(d,p) level with the PCM of chloroform S6

Figure S7. Comparison of the experimental IR and VCD spectra of Mn(III)-Cl-salen-chxn with the corresponding Boltzmann averaged IR and VCD spectra of **low-spin** at the B3LYPD3BJ/6-311++G(d,p) level with the PCM of chloroform S7

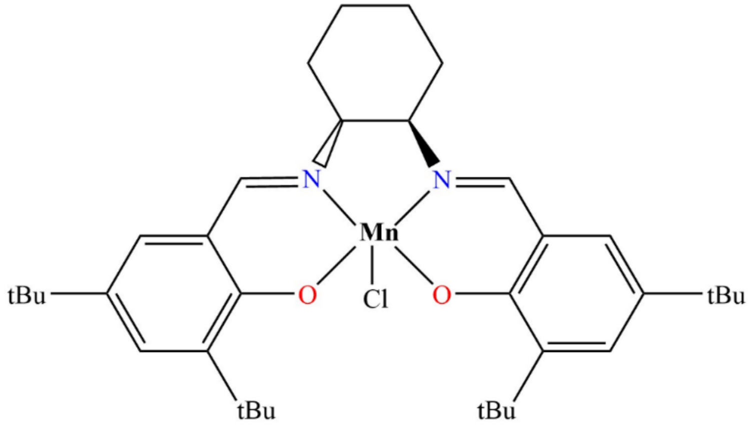

Figure S1. The molecular formula of the (*R,R*)-(+)-N,N′-Bis(3,5-di-tert-butylsalicylidene)-1,2-cyclohexane diaminomanganese(III) chloride complex, abbreviated as (*R,R*)-Mn(III)-Cl-salen-chxn. Here tBu indicates the tertbutyl group.

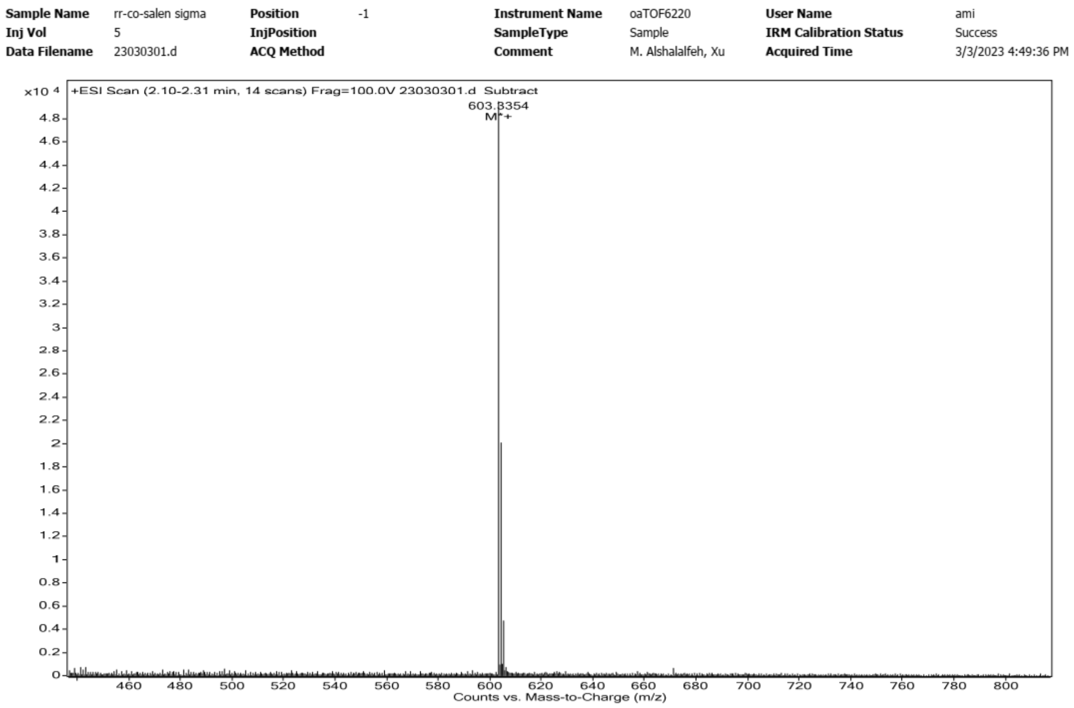

Figure S2. Experimental mass spectrum of (*R,R*)-Co(II)-salen-chxn.

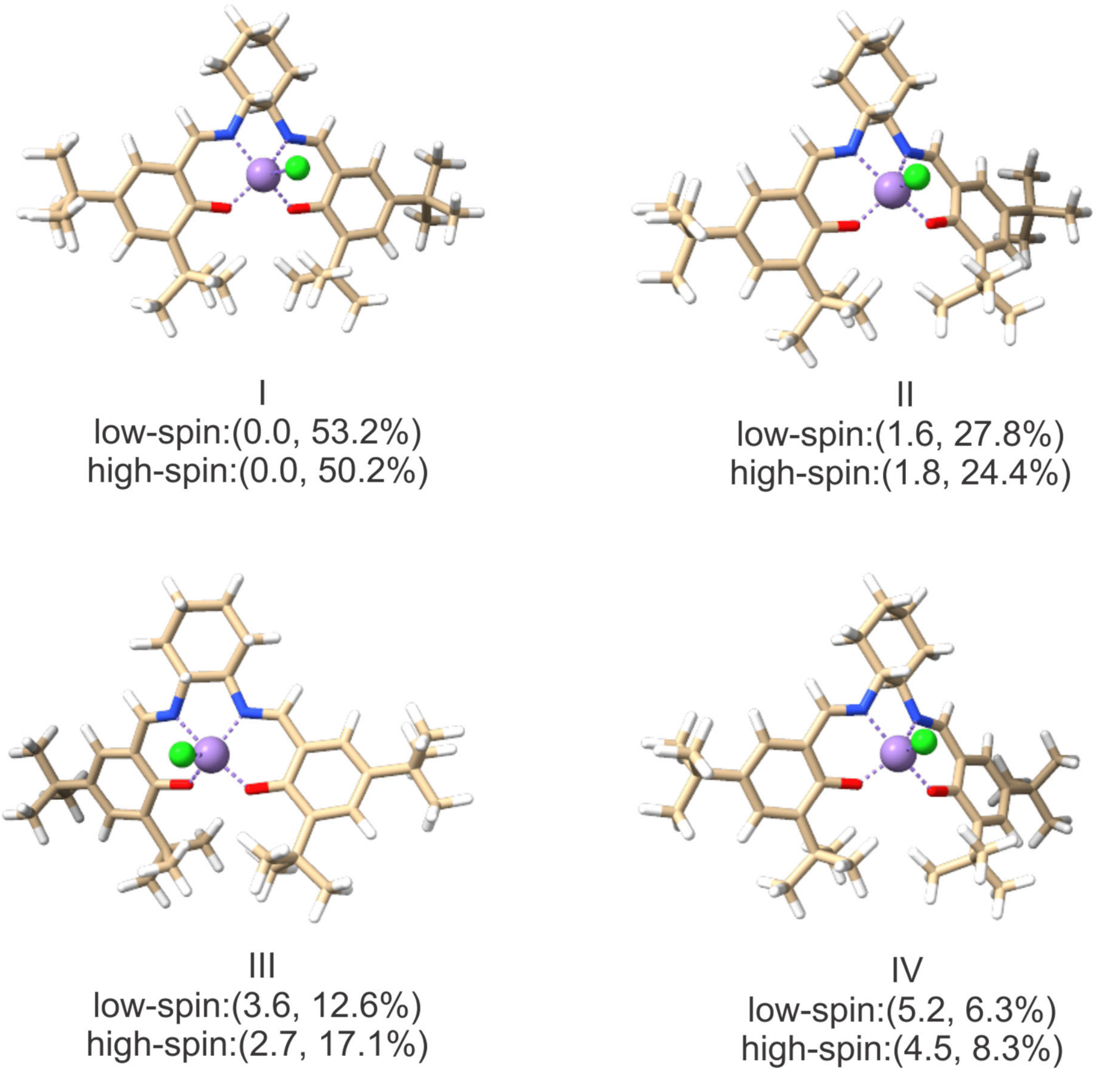

Figure S3. The optimized geometries of the four most stable Mn(III)-Cl-salen-chxn conformers at the B3LYP-D3BJ/6-311++G(d,p) level of theory with the PCM of $CDCl_3$. The relative free energies in kJ mol^{-1} at the low and high-spin of the metal complex and the Boltzmann percentage abundances at 298 K are provided in the brackets

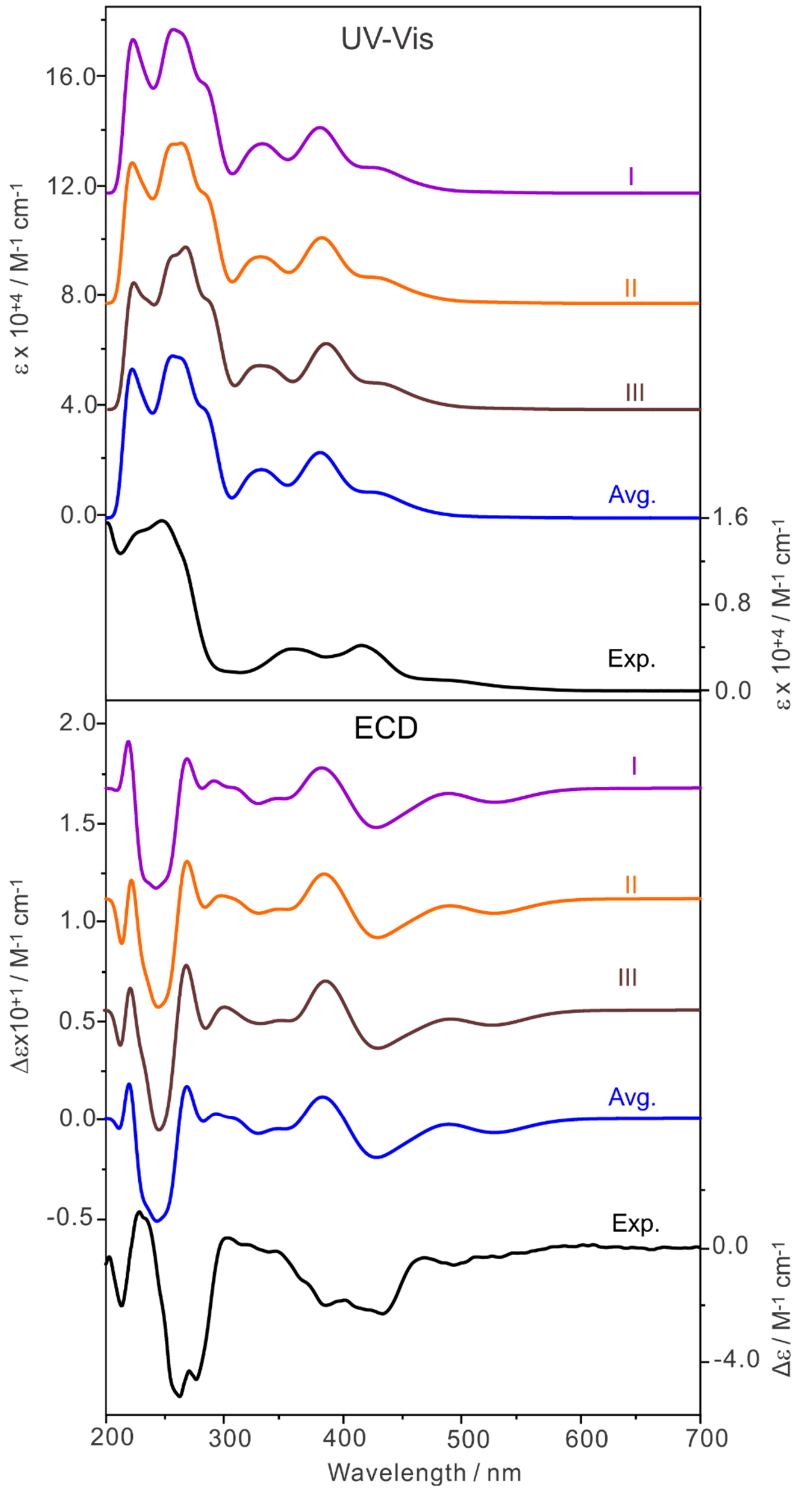

Figure S4. Comparsion of the experimental and the simulated individual and Boltzmann averaged UV-Vis (top) and ECD (bottom) spectra of Co(II)-salen-chxn (low-spin with spin multiplicity of 2) at the B3LYP-D3BJ/6-311++G(d,p)/PCM(acetonitrile) level of theory. The first 250 electronic states were included in the calculations at the level of theory indicated. The Boltzmann factors at 298 K were used.

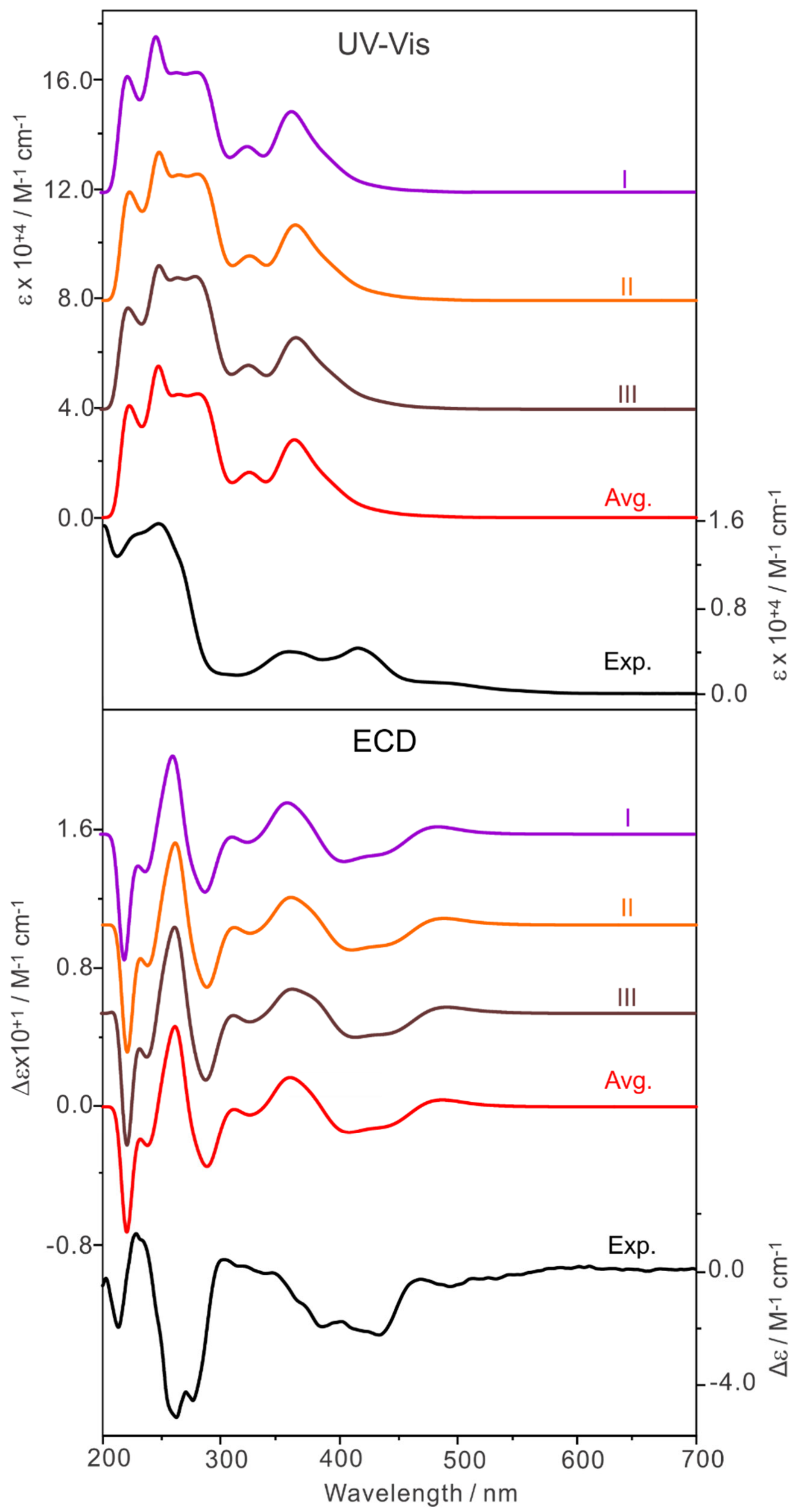

Figure S5. Comparison of the experimental and the simulated individual and Boltzmann averaged UV-Vis (top) and ECD (bottom) spectra of Co(II)-salen-chxn (high-spin, with spin multiplicity of 4) at the B3LYP-D3BJ/6-311++G(d,p)/PCM(acetonitrile) level of theory. The first 250 electronic states were included in the calculations at the level of theory indicated. The Boltzmann factors at 298 K were used.

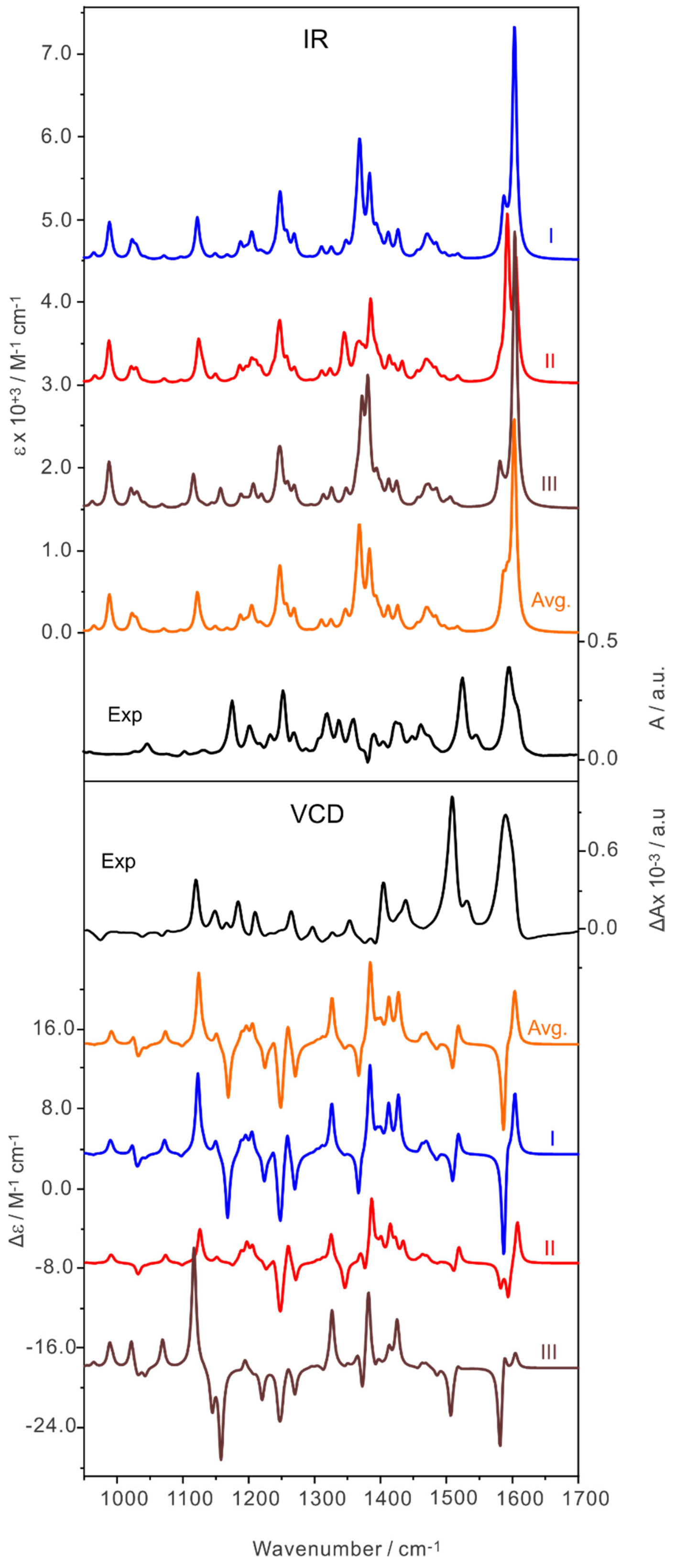

Figure S6. Comparison of the experimental IR and VCD spectra of Co(III)-salen-chxn and the simulated individual and Boltzmann average IR (top) and VCD (bottom) spectra of Co(III)-salen-chxn at charge +1 at the B3LYPD3BJ/6-311++G(d,p) level with the PCM of chloroform at 298K.

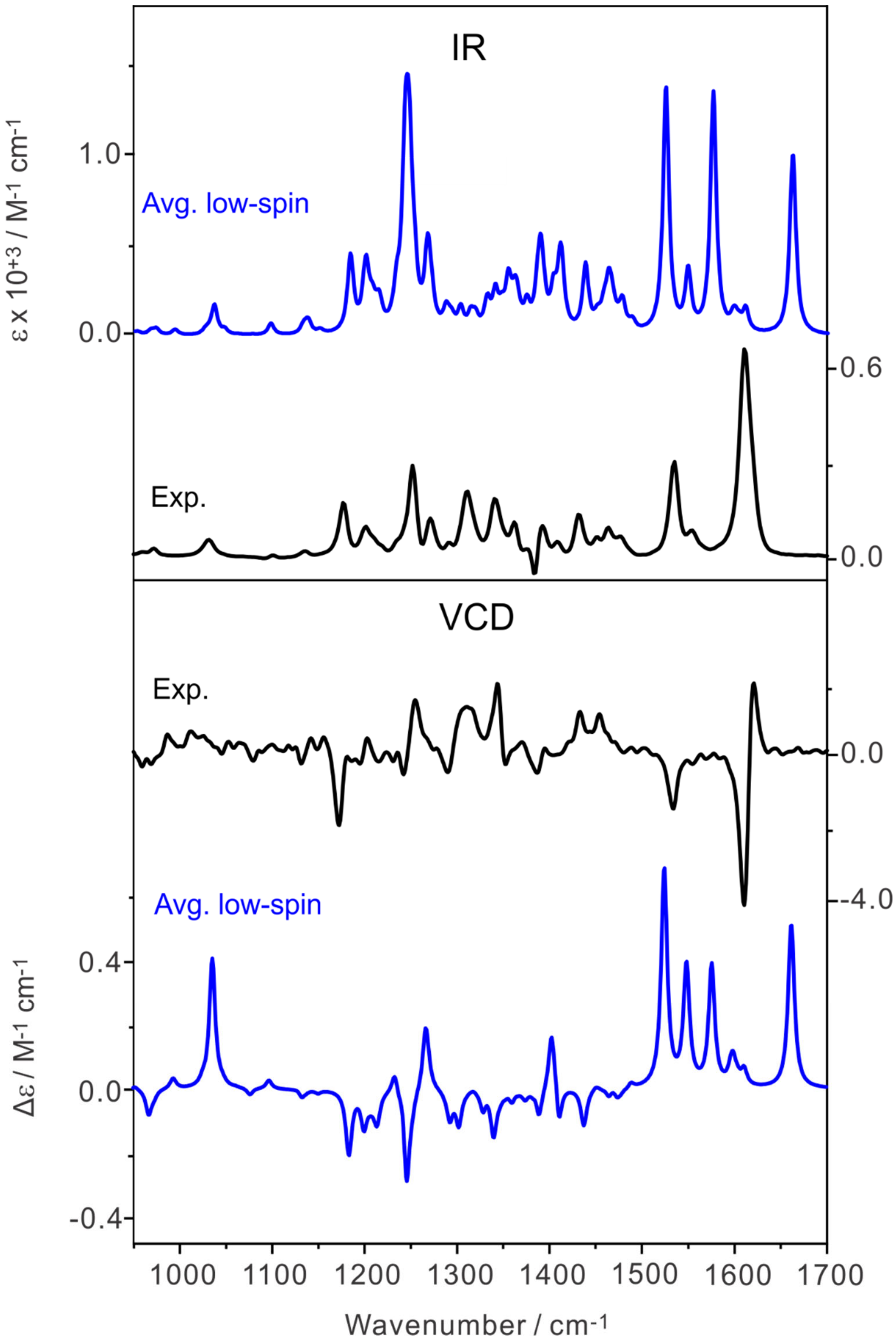

Figure S7. Comparison of the experimental IR and VCD spectra of Mn(III)-Cl-salen-chxn with the corresponding Boltzmann averaged IR and VCD spectra of the low-spin species at the B3LYPD3BJ/6-311++G(d,p) level with the PCM of chloroform at 298K.
